# A Prospective Study on the Diagnostic Value of Hyperbilirubinemia as a Predictive Factor for Appendicular Perforation in Acute Appendicitis

**DOI:** 10.7759/cureus.3214

**Published:** 2018-08-27

**Authors:** Thangadurai Ramasamy Ramu, Sakthivel Chinnakkulam Kandhasamy, Anandi Andappan, Bavani Sankar T

**Affiliations:** 1 Surgery, Government Hospital, Gobichettipalayam, IND; 2 General Surgery, Jawaharlal Institute of Postgraduate Medical Education and Research, Puducherry, IND; 3 General Surgery, Madras Medical College and Rajiv Gandhi Government General Hospital, Chennai, IND

**Keywords:** acute appendicitis, appendicular perforation, hyberbilirubinemia

## Abstract

Background

Appendicitis is one of the most common surgical emergency in general surgical practices. Early and prompt diagnosis is necessary to avoid life-threatening complications associated with it. The diagnosis is mainly clinically aided by imaging techniques. The physiological obstruction of the bile flow associated with appendicular pathology leads to hyperbilirubinemia, which can be used as a predictive factor of appendicular perforation.

Method

This prospective study was conducted in the department of general surgery in Madras Medical College and Rajiv Gandhi Government Hospital, Chennai, from January 2012 to November 2012. A total of 378 patients with the features of acute appendicitis or appendicular perforation admitted in the emergency surgical ward were included.

Results

Out of 378 of the study population, 18% had appendicular perforation and 82% had acute appendicitis. Out of 67 perforations, 60 patients have hyperbilirubinemia (90%) whereas out of 311 patients with appendicitis, only 89 (29%) of them had elevated bilirubin. Hyperbilirubinemia with a cutoff point of 0.9 mg% for appendicitis patients has a sensitivity of 89.6%, specificity of 71.4%, a positive predictive value of 27%, and a negative predictive value of 96.9%. Hyperbilirubinemia with a cutoff point of >1.3 mg% for appendicular perforation has a sensitivity of 80%, specificity of 89%, a positive predictive value of 93%, and a negative predictive value of 96%.

Conclusions

Hyperbilirubinemia with bilirubin levels more than 1.3 mg% are highly predictive of appendicular perforation and, hence, aid in prompt diagnosis. This can be combined with a clinical diagnosis and imaging for an accurate and precise diagnosis.

## Introduction

The most common emergency encountered in surgical practice is acute appendicitis. The diagnosis of any form of appendicular pathology is mainly clinical [1]. However, even in experts hands, there is a possibility of missing the diagnosis as well as the overt diagnosis. The currently available blood tests and radiological imaging can aid in diagnosis but are not very specific and not pertinent to the pathology involved [2].

Recent studies have shown that elevated bilirubin levels are associated with acute appendicitis and appendicular perforation. These studies emphasized that hyperbilirubinemia can be used as a marker for both acute appendicitis and appendicular perforation [1-3]. Most of the studies conducted were retrospective on a large scale while a few were prospective and were conducted on a small scale.

Taking the challenge to conduct a prospective study on this subject on a large scale and eliminating the bias, a step ahead to see whether elevated bilirubin levels have a predictive potential for appendicular perforation, thereby differentiating between acute appendicitis and perforation, seems fairly possible. To precisely predict the preoperative diagnosis and reduce the morbidity involved, proper plans should be made.

## Materials and methods

This prospective study was conducted in the general surgery department of Madras Medical College and Rajiv Gandhi Government Hospital, Chennai, from January 2012 to November 2012. Patients admitted with features of acute appendicitis or appendicular perforations in the emergency surgical ward were included. Institute Human Ethics Committee (IEC) approval was obtained for the study and informed consent was taken from all patients. All provisions of the Declaration of Helsinki were followed in this study. A total number of 378 patients was included, and the criteria for the selection of cases were based on clinical history, physical finding, and radiological, hematological, and biochemical investigations.

Inclusion criteria

All patients diagnosed with acute appendicitis or appendicular perforations clinically on admission were included. For both these groups, only patients who underwent surgery (both open and laparoscopic), whose intraoperative findings were recorded and followed up postoperatively, and confirmation was done by histopathological evaluation suggestive of appendicitis were included.

Exclusion criteria

Patients who have been conservatively managed for appendicitis were excluded. Patients documented to have a past history of liver disease, positive hepatitis B virus surface antigen (HBsAg), cholelithiasis, a malignancy of the hepatobiliary system, jaundice, chronic alcoholism, hemolytic disease, congenital or acquired biliary disease, and drug intake causing cholestasis were excluded. Patients less than 12 years of age and more than 75 were also excluded from the study.

Data were collected and entered in a prespecified proforma at admission and serially after that. Demographics (age, gender), complete blood count with peripheral smear examination, tests for liver function (serum total bilirubin: direct, indirect), urea and creatinine, HbsAg, and hepatitis C antibody (anti-HCV), imaging (ultrasonography), and clinical score were recorded. The normal bilirubin range in adults was taken as direct bilirubin 0.1–0.3 mg%, indirect bilirubin 0.2–0.8 mg%, and total bilirubin 0.3–1.0 mg%. Patients were diagnosed clinically, aided by imaging studies, and were taken for emergency appendicectomy by either the open or laparoscopic method.

Statistical analysis

Statistical analysis was done using SPSS version 24 for Windows (IBM Corp., Armonk, NY, US). Kolmogorov-Smirnov tests of normality tested the normality of data. Means for normally distributed data were compared using the student's test for two groups.

## Results

Age and sex distribution

In our study, out of the 378 study population, 207 were male and 171 were female, i.e., 55% were male and 45% were female. Out of the 378 study population, 18% had an appendicular perforation and 82% had acute appendicitis. A majority of the study population was between 15 and 35 years (Table 1).

**Table 1:** Age distribution

Condition	Age (Years)	Bilirubin > 1	Bilirubin < or = 1	Total
Appendicular Perforation	11-20	15	1	16
	21-30	27	3	30
	31-40	8	2	10
	41-50	6	1	7
	51-60	2	0	2
	61+	2	0	2
	Total	60	7	67
Acute Appendicitis	11-20	28	81	109
	21-30	26	81	107
	31-40	16	45	61
	41-50	12	10	22
	51-60	7	3	10
	61+	0	2	2
	Total	89	222	311

**Figure 1:**
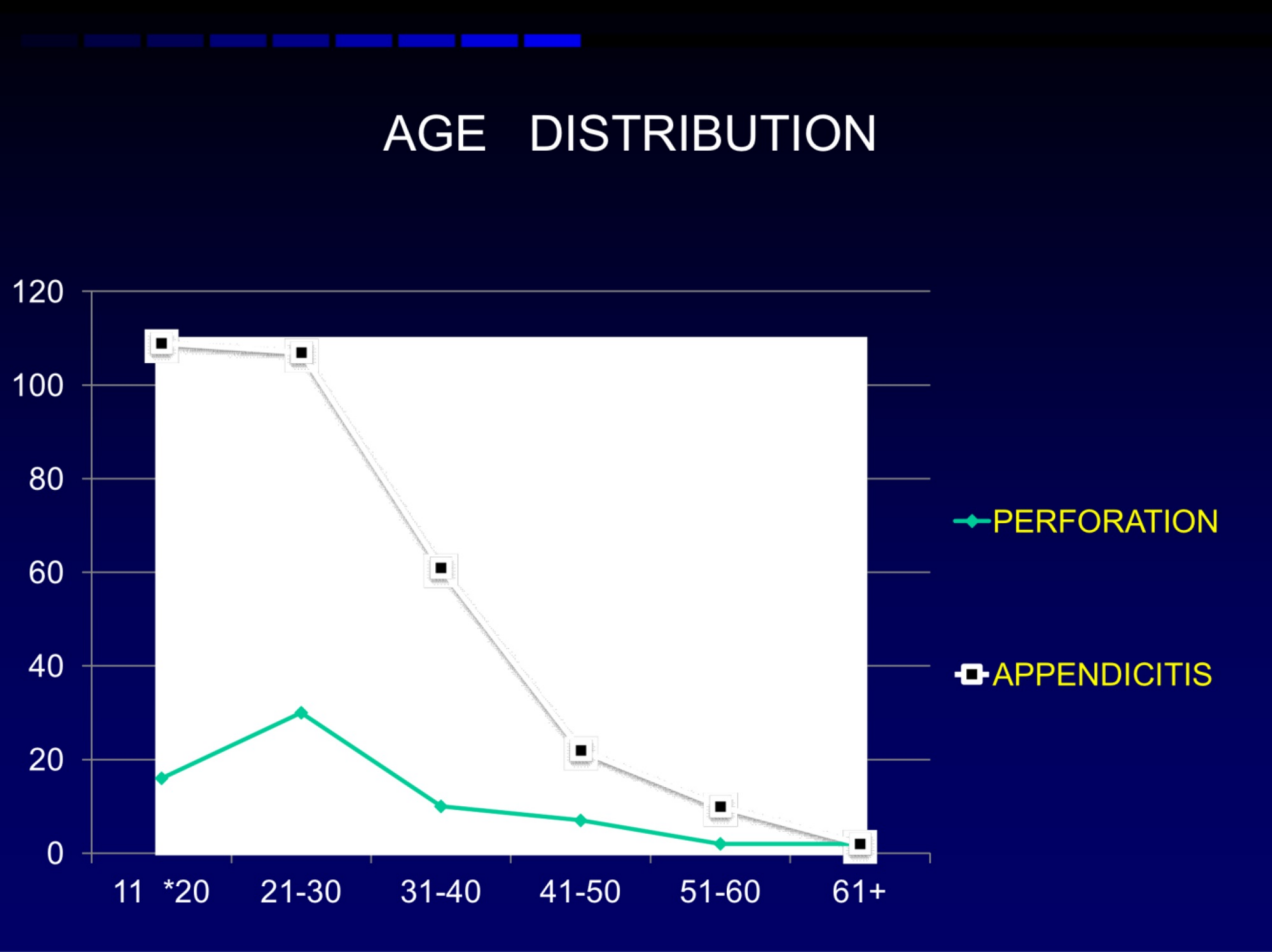
Age distribution

Most patients were in the second and third decades of life and the frequency decreased as the age advanced (Figure 1).

Distribution of bilirubin levels

**Figure 2:**
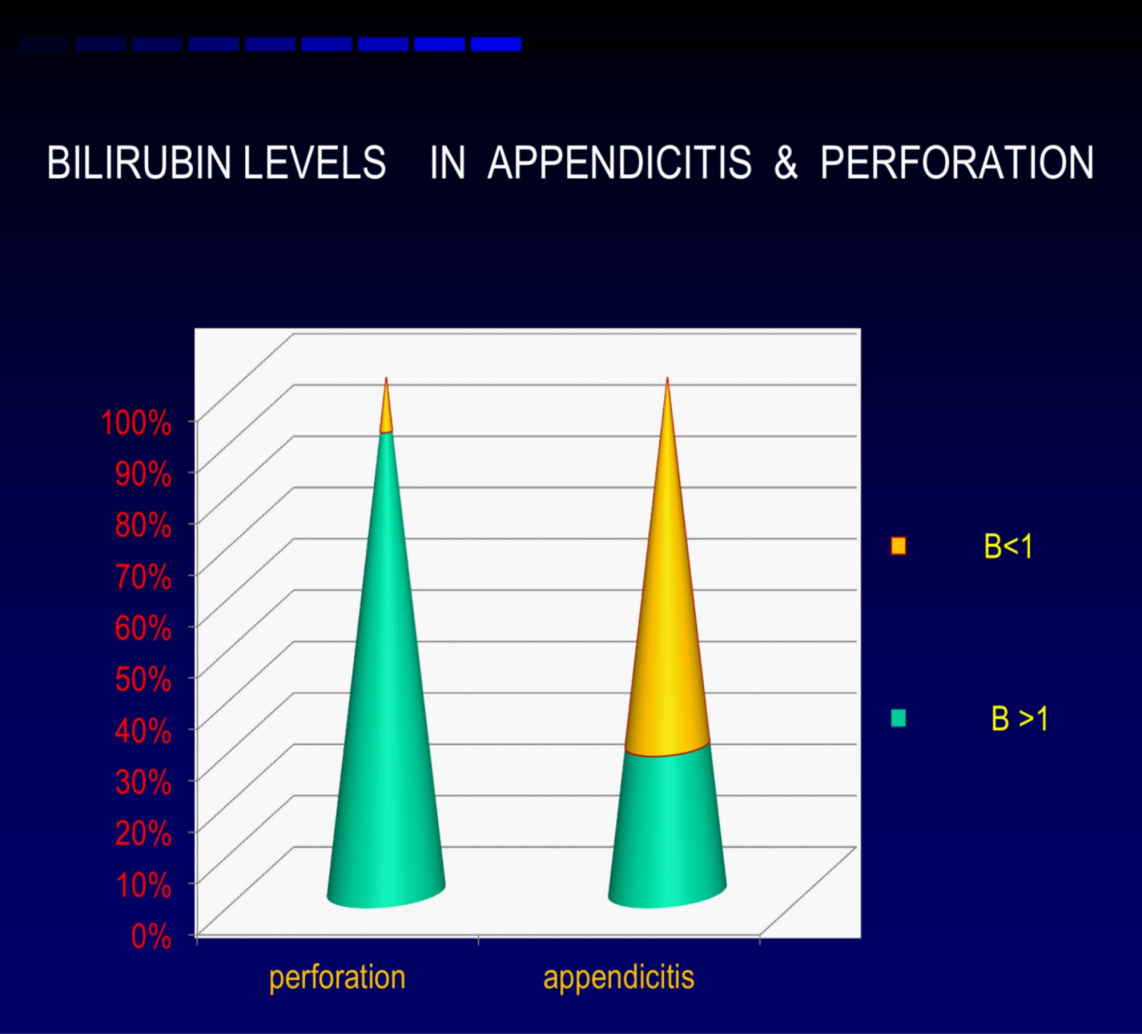
Distribution of bilirubin

Out of the 67 perforations, 60 patients had hyperbilirubinemia (90%), whereas out of 311 patients with appendicitis, only 89 (29%) of them had elevated bilirubin. These findings suggested hyperbilirubinemia was more commonly associated with appendicular perforation than with non-suppurative appendicitis, that too with a significant elevation (Table 2, Figure 2).

**Table 2:** Distribution of bilirubin levels

	Bilirubin (mg%) > 1	< or = 1	Total
Appendicular Perforation	60	7	67
Acute Appendicitis	89	222	311
Total	149	229	378

**Figure 3:**
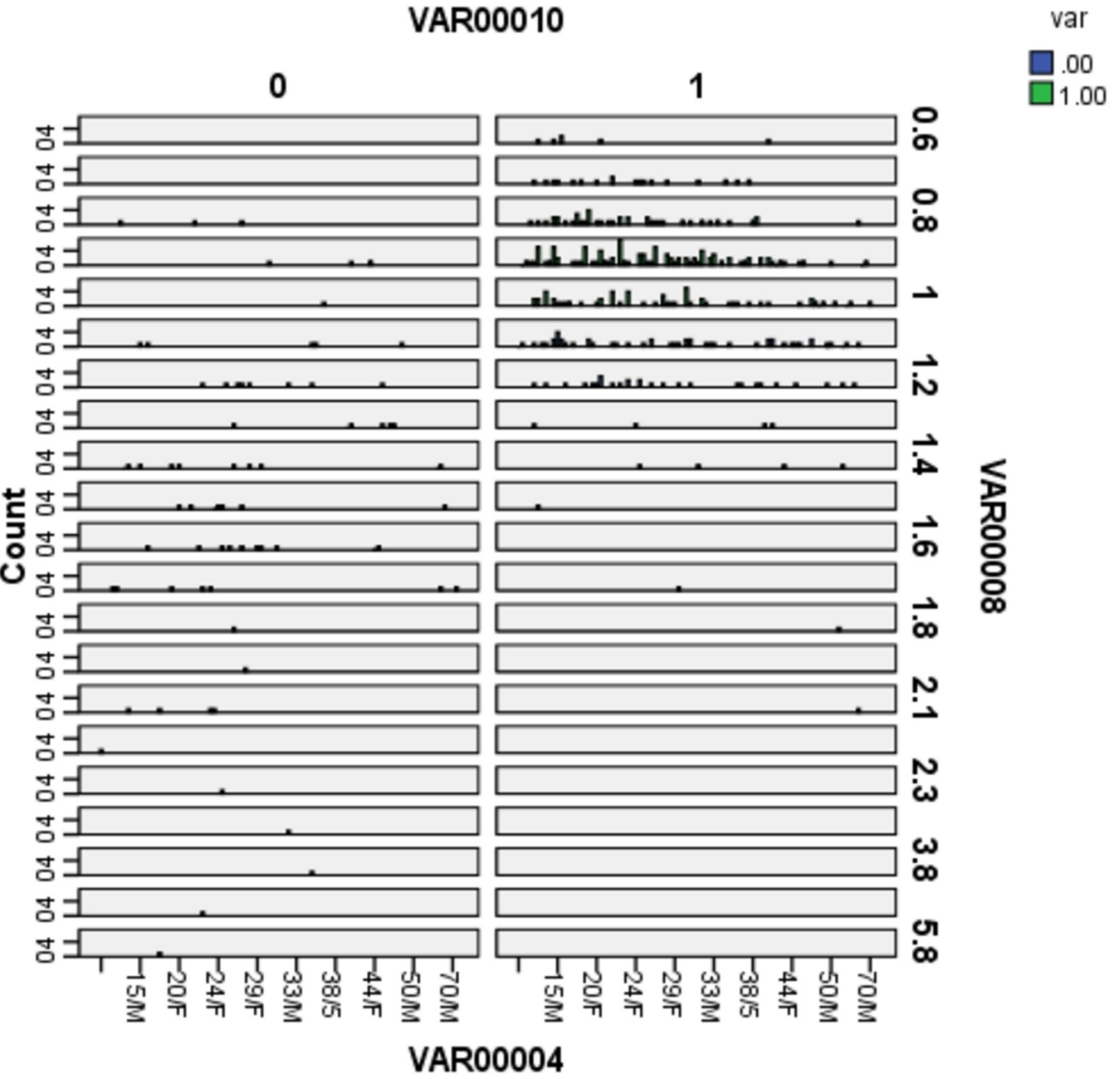
Clustering of cases: appendicular perforation (0) vs. acute appendicitis (1)

The clustering of cases in acute appendicitis occurs with corresponding bilirubin levels between 0.8 mg% and 1.2 mg%. The clustering of cases in appendicular perforations occurs with bilirubin levels corresponding to ≥ 1.3 mg%. This observation can be exploited to differentiate the patients with appendicular perforations and acute appendicitis having elevated bilirubin levels (Figure 3).

The mean value of bilirubin in appendicular perforations is 1.63 mg%, the mean value in acute appendicitis is 0.97 mg%, and the difference in the mean bilirubin levels is found to be statistically significant with p-value < 0.001. The normalization of bilirubin and alkaline phosphatase (ALP) occurs postoperatively within 48 and 72 hours (perforation cases) and 24 and 48 hrs (non-perforated cases) (Table 3).

**Table 3:** Correlation of bilirubin levels

	N	Mean Bilirubin Level (mg%)	Standard Deviation	Standard Error of Mean
Appendicular Perforation	67	1.6313	0.87216	0.10655
Acute Appendicitis	311	0.9691	0.18089	0.01026

Hyperbilirubinemia with a cutoff point of 0.9 mg% for appendicitis patients has a sensitivity of 89.6%, a specificity of 71.4%, a positive predictive value of 27%, and a negative predictive value of 96.9%. Hyperbilirubinemia with a cutoff point of >1.3 mg% for appendicular perforations has a sensitivity of 80%, a specificity of 89%, a positive predictive value of 93%, and a negative predictive value of 96%.

In a majority of the cases, direct bilirubin is much more elevated than the indirect bilirubin. Even in patients with normal total bilirubin, direct moiety is elevated (>15% of the total bilirubin). This supports the postulated physiological bile flow obstruction.

## Discussion

Whereas non-perforated acute appendicitis can be cured by an appendectomy without a long recovery period, perforated appendicitis or suppurative appendicitis can cause various complications that can result in life-threatening conditions [4]. Recent developments in the diagnosis of acute appendicitis with the assistance of radiological tools, such as ultrasonography and computed tomography (CT) have reduced the rate of negative appendicectomies. Although the reported diagnostic accuracy of ultrasonography varies depending on the patient population studied, a meta-analysis showed an overall ultrasonographic sensitivity of 85% and a specificity of 92% [5].

The diagnostic utility of ultrasonography for acute appendicitis has been emphasized and widely accepted, especially for the pediatric and pregnant patient groups [6]. Because of the development of helical CT, the effectiveness and accuracy of diagnosing appendicitis have already overcome the limitation of ultrasonography, with sensitivities of 90% to 99% and specificities of 91% to 99% [7]. However, a recent analysis by Pritchett et al. [8] showed that the increasing use of CT scanning in acute appendicitis increases the cost of care and the staying time in the emergency department and delays the time to intervene surgically. Because physical examinations and laboratory tests are still acknowledged as being of utmost importance in the diagnostic process [9], we tried to find key laboratory tests that would allow us to anticipate the severity of acute appendicitis.

Atahan et al. [10] concluded that the assessment of preoperative total bilirubin is useful for the differential diagnosis of perforated versus acute suppurative appendicitis, whereas a white blood cell (WBC) assessment is effective for diagnosing the presence versus absence of appendicitis. Symptom duration, WBCs, and total bilirubin should be used as independent parameters in the early diagnosis of appendix perforation.

A majority of our study population was between the ages of 11–30 years. These results were comparable to the study conducted by Panagiotopoulou et al., which showed that the age group 17-39 years had acute appendicitis [1] and Kumar et al. showed an age group of less than 30 years [2]. It is fascinating to see that the frequency distribution of age groups in acute appendicitis peaked at the second followed by the third decade whereas perforation peaked in the third decade followed by the second decade. In the present study, men dominated women in sex distribution. This analysis was comparable to the study conducted by Hong et al. who had a distribution of men 51.38% and women 48.61% [3].

Most patients with perforated appendicitis have hyperbilirubinemia (90%), whereas in patients with appendicitis, only 29% had elevated bilirubin. These findings were comparable to the study conducted by Kumar et al. which specified 63% versus 33%, respectively [2]. These findings suggested hyperbilirubinemia was more commonly associated with appendicular perforation than with non-suppurative appendicitis, that too with a significant elevation.

The clustering of cases in acute appendicitis occurs with corresponding bilirubin levels between 0.8 mg% and 1.2 mg%. The clustering of cases in appendicular perforations occurs with bilirubin levels corresponding to ≥1.3 mg%. These findings are similar to studies by Kumar et al. in which 39.13% of perforated appendicitis and 24.07% of non-perforated appendicitis fell in the total bilirubin range of 1-2 mg/dl. This observation can be exploited in differentiating patients with appendicular perforations and acute appendicitis who have elevated bilirubin levels [2].

In our study, the mean value of bilirubin in appendicular perforations is 1.63 mg%, which was comparable to Kumar et al., who found that more than 1.5 mg/dl was predictive of appendicular perforation [2]. Motie et al. found that bilirubin >0.85 mg/dl was the cutoff value for the prediction of perforated appendicitis [11]. Mir et al. found that increased bilirubin levels (≥1.5 mg/dl) were found to have a high positive predictive value for detecting perforated appendicitis [12]. In our study, the mean value of bilirubin in acute appendicitis is 0.97 mg%. This finding was comparable to studies by Cheekuri et al., who found that a bilirubin of 1.125 was predictive of acute appendicitis [12].

Chaudhary et al. [13] showed a rough estimation that the level of serum bilirubin was higher than 3 mg/dl in cases of gangrenous/perforated appendicitis while in cases of acute appendicitis, it was lower than 3 mg/dl (P<0.05), stating that it was predominantly isolated hyperbilirubinemia in the majority of cases.

Hyperbilirubinemia with a cutoff point between 0.9 and 1.3 mg% for acute appendicitis and >1.3 mg% for appendicular perforations was highly sensitive and predictive to differentiate between these two entities. The outcomes of our study (bilirubin 1.63 mg% in perforation versus 0.97 mg% in acute appendicitis) are slightly different from the retrospective study conducted by Bechara et al. with the mean value of all patients at 0.9 mg/dl. Those with appendiceal perforation, however, had a mean bilirubin level of 1.5 mg/dl, which was significantly higher than those with nonperforated appendicitis (p<0.05) [14]. It is prudent to keep the bilirubin level cutoff at 1.3 mg% for appendicular perforation; the only negative impact of the increase in cutoff point to 1.3 mg% is the decrease in sensitivity. To overcome this shortcoming, we combined the clinical assessment and bilirubin cutoff as >1.3 for sensitivity alone due to which sensitivity rises to 97%.

By combining the clinical diagnosis and bilirubin levels (cutoff 1.3 mg%), the detection rate of appendicular perforation rises from 82% to 97%, which is very significant, and this is possible because they are complementary to each other. This would suggest considering hyperbilirubinemia for its clinical implication in improving precision in preop diagnosis and planning.

## Conclusions

Patients with hyperbilirubinemia at a cutoff of >1.3 mg% and having clinical symptoms of appendicitis should be identified as having a higher probability of appendiceal perforation than those with normal bilirubin levels since clinical diagnosis and hyperbilirubinemia complement each other.
